# Volcanic forcing of the Lomagundi–Jatuli carbon isotope excursion

**DOI:** 10.1073/pnas.2519431122

**Published:** 2025-12-01

**Authors:** Janne Blichert-Toft, Kurt Konhauser, Baptiste Coutret, Marine Pinto, Arnaud Agranier, Abderrazzak El Albani, Francis Albarède

**Affiliations:** ^a^Laboratoire de Géologie de Lyon, CNRS UMR 5276, Ecole Normale Supérieure de Lyon, Lyon 69007, France; ^b^Department of Earth and Atmospheric Sciences, University of Alberta, Edmonton, AB T6G 2E3, Canada; ^c^Geo-Ocean, UMR6538, University of Brest, CNRS, Ifremer, Plouzané 29280, France; ^d^Institut de Chimie des Milieux et Matériaux de Poitiers, University of Poitiers, UMR-CNRS 7285-IC2MP, Poitiers 86073, France

**Keywords:** Lomagundi–Jatuli Event (LJE), carbon isotopes, volcanic outgassing, Eburnean Orogeny, Dole Effect

## Abstract

The Lomagundi–Jatuli Event (LJE), Earth’s most pronounced and prolonged positive carbon isotope excursion, followed the Great Oxidation Event and remains enigmatic in origin. We present a Pb–Pb isochron age of 2,194 ± 5 Ma for black shales from the Francevillian FB Formation, Gabon—an LJE type locality—coinciding with the emplacement of the Large Igneous Province that gave rise to the extensive Birimian–Eburnean orogenic segment. This orogeny produced very large volumes of juvenile crust in the form of oceanic plateau basalts that were eventually reworked through subduction and collisions. These processes released substantial volcanic CO_2_ into the ocean–atmosphere system, disrupting the carbon and oxygen cycles for 100 to 200 My. Volcanic outgassing likely outpaced alkalinity production from weathering, leading to an increase in the δ^13^C of sedimentary carbonates. Reanalysis of global δ^13^C–δ^18^O datasets reveals a previously unrecognized dual bimodality, attributed here to episodic volcanic degassing and subsequent oceanic carbonate saturation. Weathering of emergent crust enhanced nutrient fluxes, driving primary productivity and organic carbon burial. We further propose that volcanic CO_2_ emissions modulated the Dole Effect by shifting the balance between terrestrial and marine photosynthetic oxygen production, altering atmospheric oxygen isotope ratios independently of climate. These processes collectively drove the δ^13^C excursion of the LJE and may have fostered conditions conducive for early eukaryotic evolution.

## Significance

The Lomagundi–Jatuli Event (LJE), more than 2 billion years ago, marks a major shift in Earth’s carbon cycle, following the rise of atmospheric oxygen. Its cause, however, remains unresolved. We dated black shales from Gabon to 2.194 billion years ago, coinciding with the onset of intense volcanic activity during the Birimian–Eburnean orogeny. These eruptions released vast amounts of CO_2_, disrupting global carbon and oxygen cycles for over 100 My. By firmly linking the LJE to volcanism and juvenile crust formation, our study provides an original and integrated explanation connecting deep Earth processes to Proterozoic carbon cycling and the environmental conditions that may have set the stage for complex life. We further identify a volcanic influence on the Dole Effect. Taken together, the isotopic signatures of massive CO_2_ release, changes in ocean chemistry, enhanced nutrient fluxes, and extensive burial of organic carbon—anchored by robust chronology—account for the enigmatic isotopic anomalies of the LJE.

The unusually positive δ^13^C values observed during the Lomagundi–Jatuli Event (LJE) are commonly attributed to a major increase in organic carbon burial (1). According to this view, high primary productivity led to the preferential burial of ^12^C-enriched biomass, leaving the overlying water column enriched in ^13^C, thus shifting its δ^13^C signature to more positive values. This extensive C_org_ sequestration is thought to have contributed to a buildup of atmospheric oxygen (O_2_), as the oxidation of buried C_org_—normally consuming O_2_—was circumvented. An alternative hypothesis proposes that, in environments depleted in electron acceptors such as O_2_ and sulfate (SO4^2−^), biological productivity was instead driven by methanogenic diagenesis (2). In this scenario, microbial degradation of C_org_ within the sediment pile generated isotopically light methane (CH_4_), which escaped into the atmosphere, leaving behind seawater and sediments enriched in ^13^C. This enrichment ended with the evolution of respiration and the marginalization of methanogens (3). More recently, however, the very existence of abnormally positive δ^13^C values during the LJE has been questioned. According to yet another perspective, the atypical δ^13^C values may result from local depositional effects, where variations in carbonate sediment facies obscure any global carbon cycle signals (4, 5).

The LJE is generally considered to have followed the Great Oxidation Event (GOE) and the end of the Huronian glaciation at around 2.22 Ga (6, 7), although the precise timing of these transitions remains uncertain. The GOE, which marks the first sustained rise in atmospheric O_2_, significantly altered Earth’s redox balance and geochemical cycles, creating conditions that may have influenced the onset of the LJE. However, determining when exactly the LJE began remains challenging. Martin et al. (8) identified the oldest reliable age for the onset of the LJE as 2,209.6 ± 3.5 Ma, based on baddeleyite analysis from the Nipissing intrusion in the Huronian Supergroup, Canada (9). As for its duration, Martin et al. (8) proposed that in Fennoscandia, the LJE ended around 2,106 ± 8 Ma, although it may have persisted as late as ~2056 Ma. Robust constraints on the exact timing of the onset and termination of the LJE remain scarce and somewhat inconclusive; nevertheless, the age range of 2210 to 2056 Ma proposed by Martin et al. is widely cited and adopted in the literature. Tight time constraints are essential for understanding whether the LJE was a singular event or part of a broader tectonic and geochemical evolution that included the GOE but, until this study, a precise timing of the LJE has been missing (*SI Appendix*, section 1).

The present study has three core objectives: 1) provide high-precision Pb–Pb geochronology of the FB Formation in order to better constrain the start and duration of the LJE; 2) present isotopic evidence in support of superplume activity as the primary driver of the LJE; and 3) assess the geochemical consequences of superplume emplacement for the Proterozoic carbon cycle and the buffering capacity of seawater.

## Geological Context and Study Site.

**Fig. 1.**
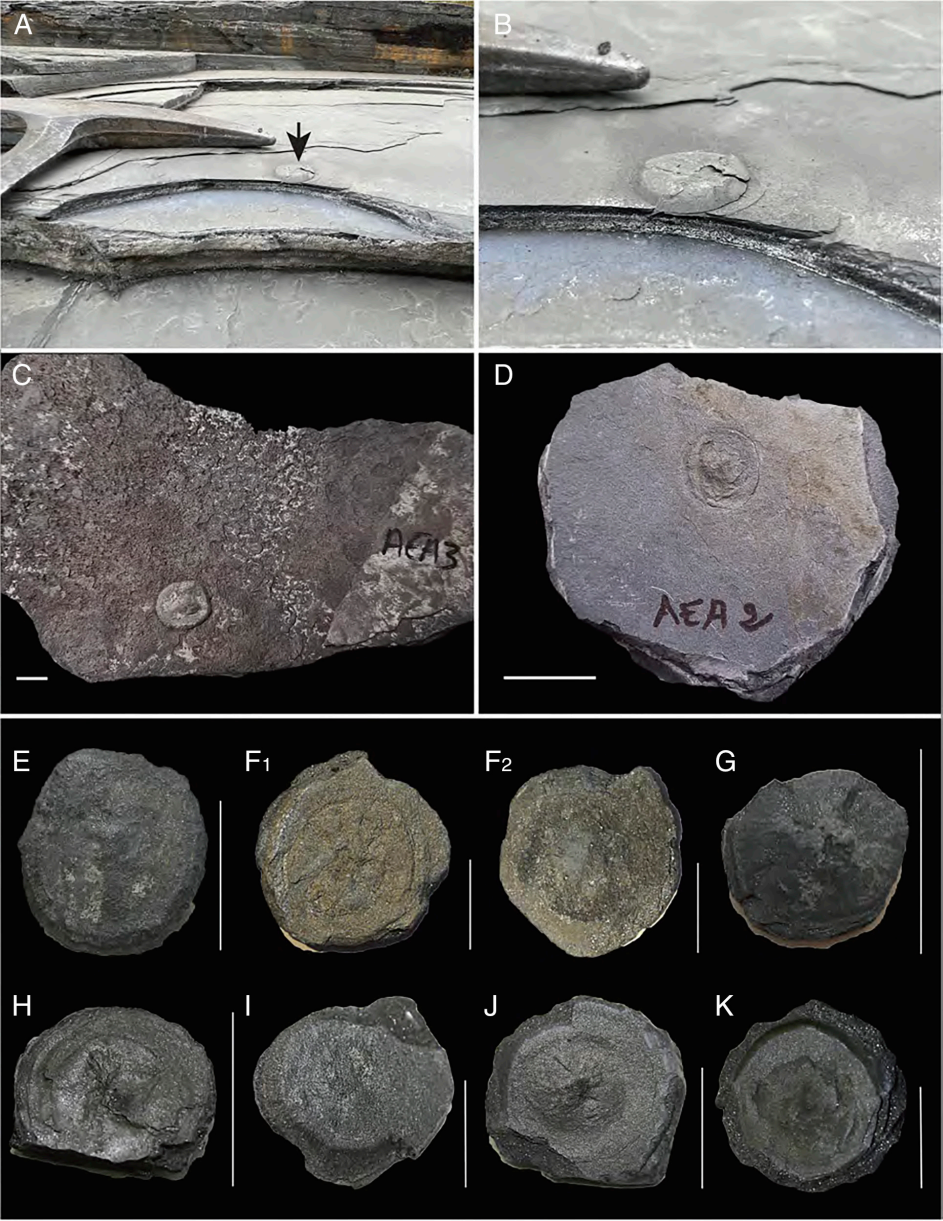
Outcrop photos and plate photographs of the lenticular forms and their Francevillian FB host black shales. The samples are those analyzed in this study. (*A* and *B*) In situ lenticular form (black arrow) outcropping in the Moulende quarry in Gabon (bedding plane view). The horizontally laminated black shales are interbedded with thin, silty sandstone layers hosting the lenticular forms (black arrow showing one). (*B*) is a close-up of (*A*) (see ref. 14 for additional information). (*C* and *D*) Samples of lenticular forms embedded in their black shale host rocks. (*E*–*K*) Lenticular forms excavated from their black shale host rocks. All lenticular forms were photographed from a top view, except for (F_2_), which represents the bottom face of (F_1_). The overall morphology of the lenticular forms is lenticular (hence the descriptive name “lenticular forms”), dominated by an oval to circular outline displaying a distinctive brim (*B*–*D*, *F*, and *K*) and/or a central convex protuberance (knob) (*C*, *F_1_*, *G*, *J*, and *K*). In some cases, a rounded bulge is preserved within the circular outline (*G*, *I*, and *K*). All white scale bars are 2 cm. The lenticular forms were carefully removed from their black shale host rocks using an air scribe, or air pen, from ZOIC PalaeoTech Ltd.

One of the most important LJE sedimentary sequences is the Francevillian Group in southeastern Gabon, which provides a well-preserved record of this period. This sequence is subdivided into five Formations (FA–FE) (10, 11), all of which are essentially unmetamorphosed. The FA Formation, at the base, consists of fluviatile sandstones and conglomerates and hosts the famous Oklo natural reactors (12), a unique example of naturally occurring nuclear fission. The overlying FB Formation is composed of sandstones, dolostones, and black shales, the latter containing 0.5 to 15 wt.% total organic carbon (13). This formation also hosts centimeter-sized, nonpyritized lenticular forms (Fig. 1), interpreted by some as the oldest fossil eukaryotes (14), though their biological origin remains debated (15, 16). The FC Formation consists of dolostones, cherts, and jasper, while the upper FD and FE Formations are dominated by black shales and sandstones.

**Fig. 2.**
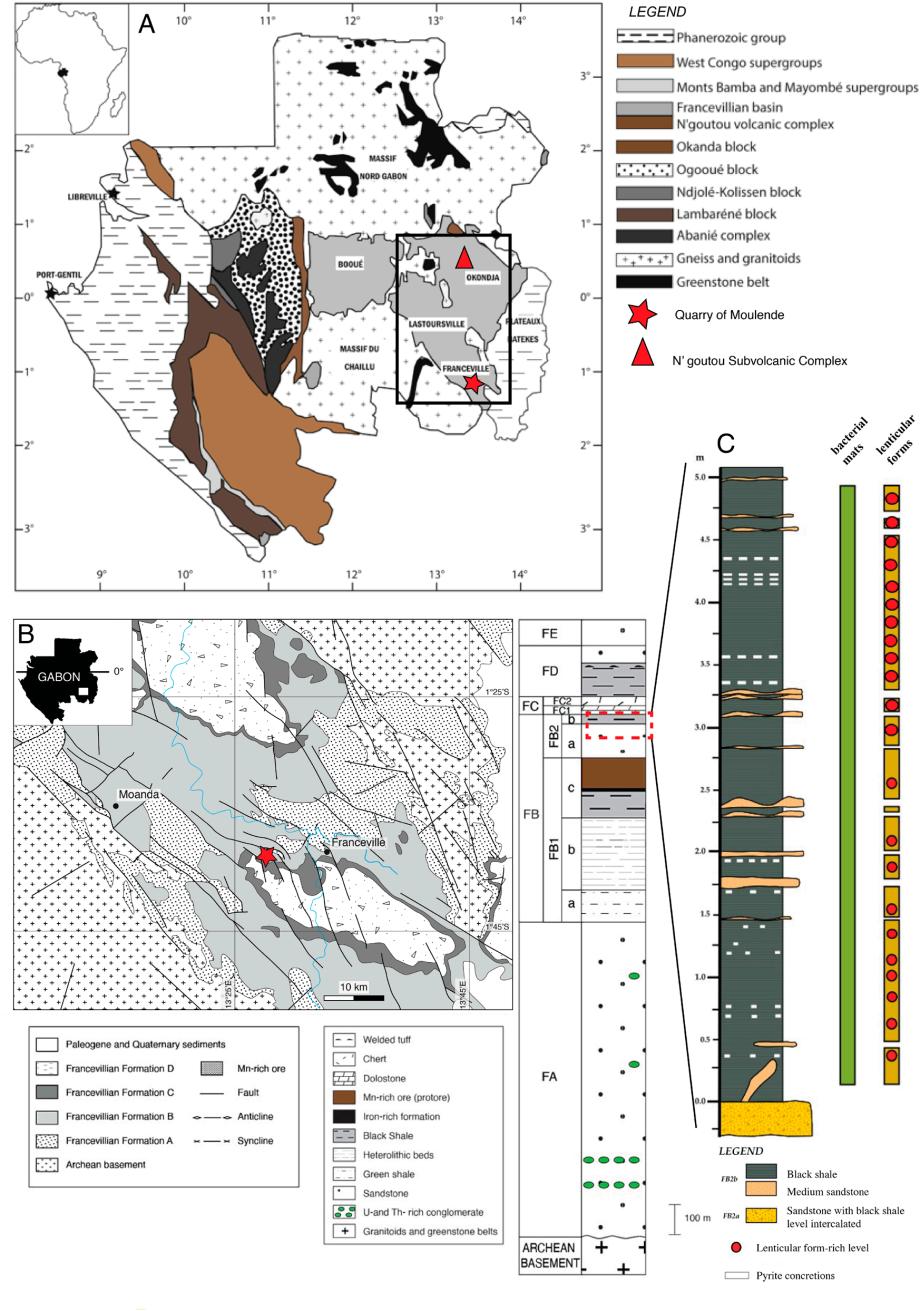
(*A*) Geological map of Gabon showing the locations of the Moulende quarry near Franceville and the N’Goutou subvolcanic complex within the Franceville Basin. (*B*) Geological map of the Franceville Basin with the studied outcrops in the Moulende quarry (orange square). The Francevillian Series is subdivided into five formations (FA to FE). The dotted red square marks the FB2a–FB2b black shale subunits and the location of the detailed lithology column (C) from the Moulendé quarry, where the lenticular form specimens were collected. Geological map adapted from Bouton et al. (2009). (*C*) Detailed lithology of the FB2 subunit in the Moulendé quarry. Red dots mark the stratigraphic levels containing lenticular specimens.

Despite its potential importance for understanding microbial evolution during the Paleoproterozoic, the Francevillian Group (Fig. 2) has not yet been directly and precisely dated. The most robust indirect age constraint comes from the N’goutou subvolcanic ring-complex, next to Okondja (17), where granite NG12, with a Pb–Pb age of 2,191 ± 13 Ma, intrudes the FA and FB Formations, providing a minimum age for the deposition of the FB Formation. The Moulendé quarry, next to Franceville, 160 km south of the N’goutou intrusion, presents a unique opportunity to obtain a precise geochronological constraint on the local LJE, contributing to broader efforts to resolve the timing and drivers of this major carbon cycle perturbation.

## Results

### A Precise 2.19 Ga Age for the FB Formation.

**Fig. 3.**
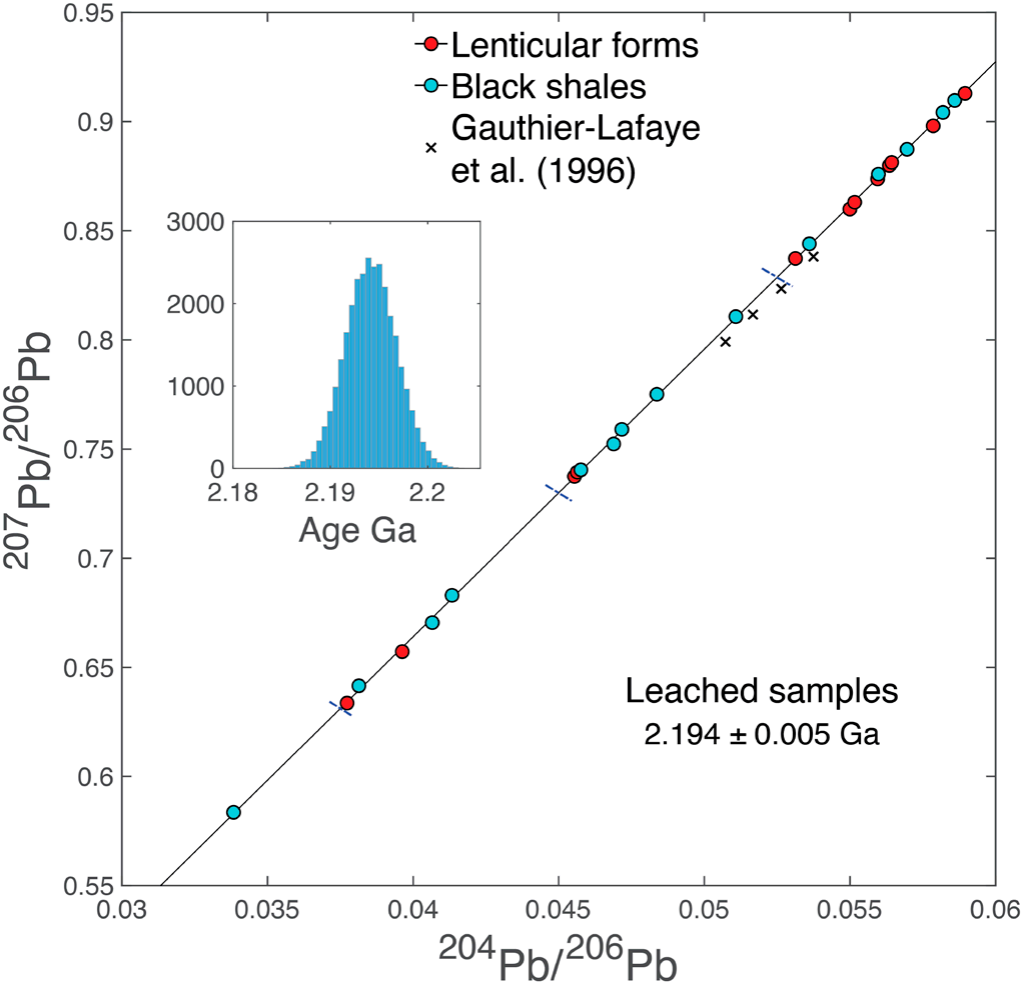
Inverse isochron plot of Pb isotopes on HCl-leached lenticular forms and host black shales from the FB Formation of the Francevillian series. The original set of data were perturbed (statistically speaking) by normal random deviates reflecting the covariance matrix of the measurements and the age calculated by averaging 30,000 Monte-Carlo runs (*Inset*). The crosses represent the Pb isotope data on leached black shales published by Gauthier-Lafaye et al. (18). The differences (offset with respect to the isochron) of the data by Gauthier-Lafaye et al. (18) relative to the data of this study reflect the evolution of the Pb isotopic reference values over the last several decades as a result of increasingly accurate and precise isotopic measurement abilities (notably by triple-spike thermal ionization mass spectrometry and MC-ICP-MS). Note the large range in Pb isotope compositions of the present samples compared to the small range measured by Gauthier-Lafaye et al. (18). The blue segments represent ±1‰ per mass unit of mass-dependent isotope fractionation due to natural and analytical processes among Pb compounds or between Pb(II) and Pb(IV). The uncertainty on the age is given prior to multiplication by MSWD½. The R2 of the isochron is 0.9999.

Dating the units from the Francevillian Group has been challenging due to isotopic scatter and sample contamination (*SI Appendix*, section 1). Lead–lead data were observed to scatter by both Gauthier-Lafaye et al. (18) and El Albani et al. (14). For the latter study, bypassing sample acid leaching was required to avoid mass fractionation of zinc isotopes, the main objective of El Albani et al.’s (14) study, thereby preventing labile Pb components, and in particular contamination from the water table, to be removed. By contrast, the new set of Francevillian samples from the FB Formation analyzed here underwent aggressive leaching with hot 6 M distilled HCl prior to sample digestion and Pb separation chemistry. An age of 2,194 ± 5 Ma was obtained from the inverse Pb–Pb isochron of Fig. 3 by regressing 30,000 Monte-Carlo models, in which each point was affected by normal deviates consistent with the analytical uncertainties (*SI Appendix*, section 2). Although the R^2^ factor, which measures the proportion of variance accounted for by the linear regression model, is near-perfect at 0.9999, the value of the mean-squared weighted deviation (MSWD) is about 22, which reflects the high precision of each individual sample analysis (Dataset S1). Despite the widespread use of statistical criteria generated by the Isoplot software (19), we chose to de-emphasize the significance of the MSWD for two key reasons. Unlike other chronometers such as ^147^Sm–^143^Nd, Pb isotopic ratios are not corrected for instrumental mass bias using an internal normalization; instead, they rely on an external reference standard. In addition, natural isotope fractionation at low temperatures in groundwater, occurring among Pb compounds, hydroxides, halides, and carbonates (20) and during acid leaching of the samples (21), can induce isotopic shifts that are not captured by MSWD statistics or modeled by Isoplot. As a consequence, the statistical significance of the MSWD, particularly for Pb–Pb isochrons, should be interpreted with caution.

The Pb–Pb age of 2,194 ± 5 Ma obtained in this study for the FB Formation and the Pb–Pb age of 2,191 ± 13 Ma obtained on zircons from the N’goutou subvolcanic ring-complex, which crosscuts the lateral equivalent of the FB Formation some 150 km away (17), are statistically indistinguishable (*t* = 0.43, *P* = 0.67), confirming that they cannot be resolved within the quoted 2σ uncertainties. We therefore conclude that the 2,194 ± 5 Ma age of the FB Formation is now robustly defined.

### Chronological Coherence.

The age of the FB Formation as determined in this study renders the geological context of the FB Formation particularly intriguing because its age, which was poorly constrained until now, coincides with the Birimian–Eburnean orogenic event lasting from ca. 2.27 to ca. 1.96 Ga (22, 23). The Birimian refers primarily to Paleoproterozoic rocks (~2.2 to 2.1 billion years ago). It began with voluminous subaerial basaltic eruptions, identified by geochemical evidence as a unique episode of juvenile crust formation in West Africa and Guyana, followed by volcano-detrital successions and the emplacement of orogenic magmas. In terms of lateral extent, this event has no known parallel in Earth’s history. The Eburnean refers to the subsequent collisional and deformational stage that mostly recycled the earlier Birimian crust. In addition to the Birimian–Eburnean superplume event, other Large Igneous Provinces (LIPs) formed at that time in Earth history (24).

## Discussion

### Volcanic Activity and the Making of New Crust.

**Fig. 4.**
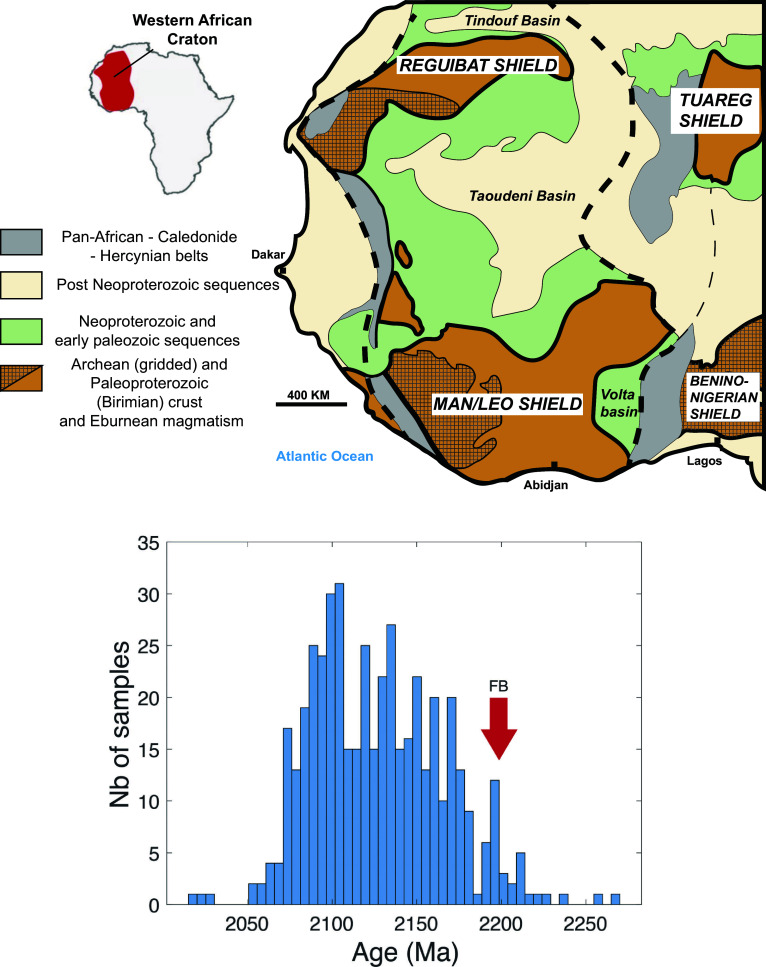
The FB layers are contemporaneous with the onset of the Birimian magmatic activity on the WAC and South America. (*Top*) outline of the WAC sketched from Baratoux et al. (25). Note that the Taoudeni basin is assumed to be underlain by Archean and Paleoproterozoic terranes. (*Bottom*) Histogram of the U-Pb zircon ages of Birimian–Eburnean magmatic rocks compiled by Grenholm et al. (23). The red arrow shows the age obtained in the present work for the FB formation (Fig. 3).

The West African Craton (WAC) covers most of West Africa (Fig. 4, *Top*). Anchored by two Archean nuclei—the Man shield and the Reguibat ridge—it is largely made up of Paleoproterozoic terranes, known as the Birimian–Eburnean, which form both its northern and southern sections and serve as the basement to extensive Neoproterozoic–Phanerozoic sedimentary basins, notably the Taoudeni basin.

The WAC Birimian terranes cover approximately 0.85 ×10^6^ km^2^, but their extension below the Taoudeni basin brings this surface area to 2.8 × 10^6^ km^2^. They bear all the geochemical signatures of juvenile crust. Although their juvenile nature is widely accepted, their geodynamic origin is still debated, with competing interpretations pointing to oceanic plateaus, back-arc basins, or intraoceanic arcs. Oceanic plateaus, which are LIPs emplaced on oceanic crust, are inherently short-lived, persisting on the seafloor for only a few tens of millions of years before entering subduction or colliding with continental margins. The presence of magmas with orogenic signatures does not preclude a protolith derived from such oceanic plateaus, nor their role in generating juvenile crust (26). A simplified timeline adapted from ref. Grenholm et al. (23) is provided in Table 1.

**Table 1.** A Birimian–Eburnean Orogeny timeline (2270–1960 Ma, adapted from ref. 23)

Age (Ma)	Phase	Dominant source	Material emitted
2270 to 2160	Early crust formation (arc, oceanic plateaus)	Juvenile mantle	Tholeiitic/calc-alkaline volcanics
2160 to 2120	Early collision	Mixed (juvenile + evolved)	Granitoids, volcanic-sedimentary
2120 to 2050	Peak collision (Eburnean)	Evolved crust + arc	Granites, sediments, metamorphic rocks
2050 to 1960	Postcollisional	Recycled crust	Late granites, molasse-type sediments

The juvenile features are the following: i) The chondrite-normalized rare-earth element distributions of the erupted magmas are flat (22, 27, 28). ii) During the 2270-2160 Ma period, the Birimian basalts conspicuously lack the niobium anomalies that are the geochemical hallmark of continental crust and arc magma environments (23, 28–31). iii) The strontium and neodymium isotopic compositions indicate that large volumes of basaltic protocrust were rapidly extracted from fertile mantle over a few tens of million years, with a remarkably short crustal history for the felsic volcanics and recycled sediments (32). iv) Titanium is incompatible during magma differentiation (22, 27, 31), which is uncommon in orogenic magmas (33), and v) volcanogenic massive sulfides are common.

Fig. 4, *Bottom* shows an age histogram of U-Pb ages for Birimian magmatic rocks, both intrusive and extrusive, compiled by Grenholm et al. (23). The onset of magmatic eruptions at ~2200 Ma is broadly consistent with both the end of the GOE and Huronian glaciations. Arc tectonics and multiple collisions of island arcs undoubtedly took place, but these events lagged behind the emplacement of plateau basalts by several tens of million years (23, 32). Within less than 100 Ma, the Birimian orogeny generated massive amounts of plateau basalts, adding more than 3 × 10^6^ km^2^ of juvenile crust to West Africa alone (2% of the modern continental surface area) as well as generating large quantities of new crust in the Guyana shield, the Sao Francisco craton in Brazil, and Uruguay (30, 34, 35).

### Ocean Chemistry Disruption and Isotope Bimodality.

There is currently no consensus on whether the LJE should be considered a global event. Hodgskiss (5) demonstrated a striking bimodal distribution of δ^13^C values in Paleoproterozoic carbonates, distinguishing between LJE and non-LJE samples, with deep-water carbonates maintaining a baseline of ~0‰ throughout the event. Prave et al. (4) and Hodgskiss (5) proposed that the unusually high δ^13^C values of the LJE correspond to nearshore-marine and coastal-evaporitic environments, but that the LJE was nevertheless inferred to be occurring on a global scale. However, we contend that the fundamental premise of the facies-based interpretation is not supported by the available δ^13^C and δ^18^O data. The notion of facies control also raises the compelling question of why the LJE would represent such a singular event in Earth history. Local environmental conditions generally vary with changes in sea level, storm frequency and intensity, and near-shore productivity, making it unlikely that facies alone can explain the full range of isotopic variations.

**Fig. 5.**
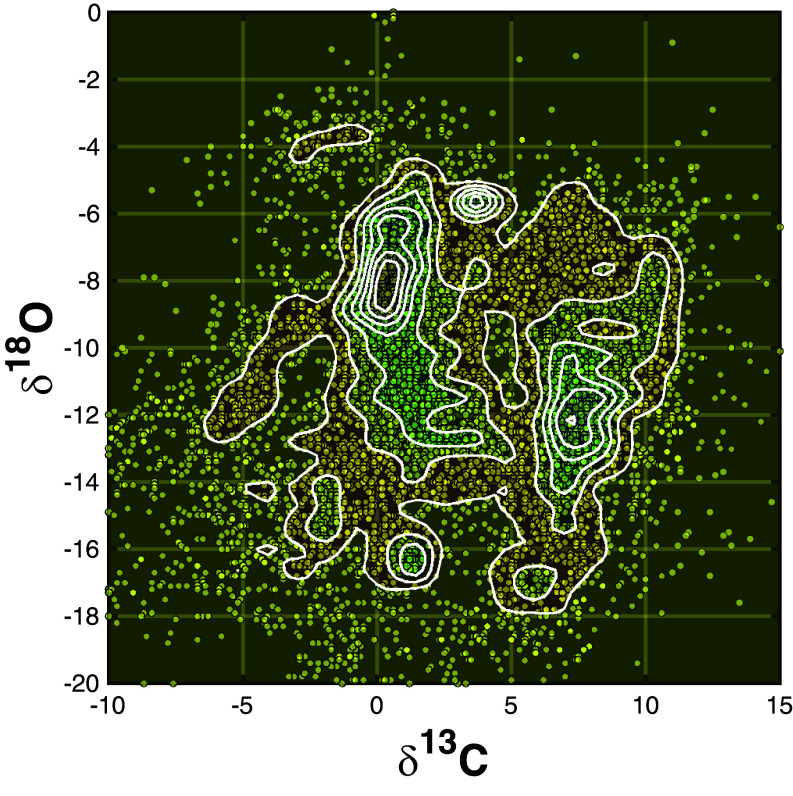
Two-dimensional density plot of δ^18^O versus δ^13^C in carbonates. Only greenschist facies or lower metamorphic grade from Hodgskiss’ (5) database are considered. This figure corresponds to Fig. 5 of these authors, but with overlain lines indicating equal sample density. Bimodality, observed by Hodgskiss (5) for δ^13^C, is also present for δ^18^O. The high δ^13^C values defining the LJE correspond to low δ^18^O.

A reanalysis of Hodgskiss’ (5) dataset, incorporating both δ^13^C and δ^18^O values, reveals a so-far undetected correlated bimodality of both isotopic systems over hundreds of millions of years. Although the density plot in Fig. 5 is influenced to some extent by variations in sampling intensity across localities, the positions of the maximum density peaks demonstrate that the highest δ^13^C values coincide with the lowest δ^18^O. This relationship argues strongly against a diagenetic overprint (36). While seawater and sedimentary carbonates may have undergone ^16^O depletion due to extensive glaciation during the Huronian ice ages (7)—potentially by 4‰, similar to the decline observed in the modern ocean over the past 50 My (37)—this explanation does not account for the matching episodes of distinctive ^13^C enrichment recorded during the LJE. Importantly, the fourth and final Huronian glaciation, the Upper Timeball Hill glaciation in Transvaal, South Africa [2260-2250 Ma, (38)], seems to coincide with the eruption of the earliest magmatic rocks of the Birimian episode.

**Fig. 6.**
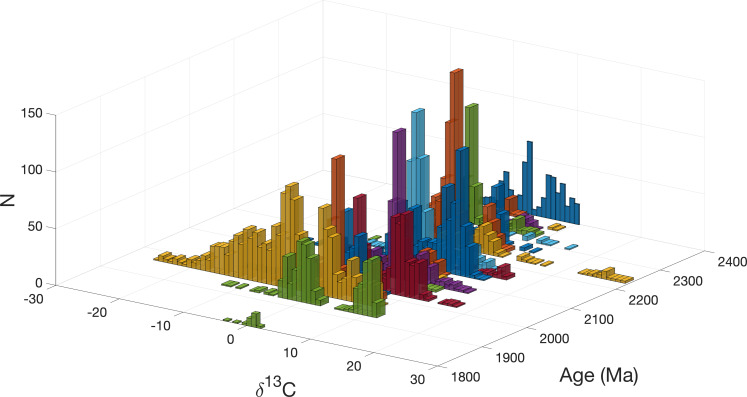
Histograms of carbonate δ^13^C across different time slices from the GOE (~2420 Ma) to 1900 Ma. The data, sourced from Hodgskiss’ (5) database, have been filtered based on metamorphic grade. Each sample *i* was evaluated by checking whether *T* falls within the reported minimum and maximum age range. Specifically, we assessed the sign of the product [min (*T_i_*) − *T*] × [max (*T_i_*) − *T*] excluding samples where the result was positive. The uncertainties in these age limits had a negligible effect. Colors are used solely to enhance readability and do not carry any specific inherent meaning.

A crucial observation that comes out of plotting δ^13^C versus δ^18^O for the duration of the LJE is that for most of the time slices, the δ^13^C distributions remain strongly bimodal (Fig. 6) with one value at 0‰ and another around +9‰ (Fig. 4). Such a distinctive global bimodality is difficult to reconcile with simple meter-scale variations in local depositional environments. In modern oceans, CH_4_ forms during early diagenesis over time scales of ~10^3^ y (39). While carbon isotope exchange occurs extensively between muds and fluids during diagenesis, this process should produce a mixed carbon signature, contradicting the strong δ^13^C bimodality observed in this study. In the absence of global glaciations during the LJE and the improbability of methanogenesis driving carbon isotope systematics for hundreds of millions of years, the observed bimodality requires another explanation.

A different approach based on the unprecedented volcanism at that time may offer a new framework for understanding the δ^13^C excursions as well as the coupled dual δ^13^C-δ^18^O bimodality associated with the LJE. The Birimian superplume, which erupted at just this time, would discharge into the atmosphere and the ocean huge amounts of mantle-derived CO_2_ with δ^13^C of about –5 to –7‰. This release of carbon would have had two major effects: 1) increased acidification of bulk seawater and 2) enhanced chemical weathering of newly emergent crust.

### Carbon Cycle Feedbacks and Buffering Limits.

A possible link between the Birimian superplume event and the LJE deserves closer investigation. The immense volumes of lava erupted during the Birimian–Eburnian orogeny represent one of the largest and most massive episodes of mantle melting recorded in Earth’s history. If partial melting of plume-derived mantle produced ~3 × 10^6^ km^2^ of juvenile lithosphere (40), the amount of CO_2_ released to the atmosphere would have reached (1 × 10^−3^ g) × (3 × 10^6^ km^2^ × 100 km) × (3.3 g/cm^3^) = 10^6^ PgC. Assuming a carbon concentration of 0.25% in a 30 km-thick crustal section (41), this would yield a comparable estimate of 0.7 × 10^6^ PgC, corresponding to roughly 10^6^ PgC of carbon, or up to 25 times the amount of modern oceanic dissolved inorganic carbon (DIC) (38,000 PgC) (42). For reference, the modern atmosphere holds approximately 900 PgC. Such a huge outburst need not be instantaneous; what matters is that the rate of mantle-derived CO_2_ production by magmatic activity exceeds the rate at which it is neutralized by the riverine flux of alkalinity. How long would it take for runoff to neutralize such a massive eruption of CO_2_?

Alkalinity (Alk) allows the ocean to buffer CO_2_ by neutralizing the acid it forms, but this buffering works only while DIC remains lower than Alk (43). At high alkalinity, most DIC is present as bicarbonate (${\mathrm{HCO}}_{3}^{-}$), but once Alk is depleted through carbonate precipitation, DIC shifts mainly to dissolved CO_2_ (H_2_CO_3_), with important isotopic consequences as discussed below.

When atmospheric CO_2_ rises—for example, after major volcanism—more CO_2_ dissolves in the surface ocean, but uptake is limited by the carbonate buffer, expressed by the Revelle factor (≈10 in surface waters). This means a 10% rise in pCO_2_ increases DIC by only ~1%. As DIC builds up and approaches Alk, carbonate (${\mathrm{CO}}_{3}^{2-}$) ions decline, pH drops, and the ocean loses its capacity to absorb additional CO_2_.

The ocean then shifts from absorbing CO_2_ to releasing it. This effect is further amplified by the formation of carbonate minerals (such as CaCO_3_), which releases about 0.6 mol of CO_2_ per mol of carbonate when they precipitate (44–46) according to the reaction:

[1] $${\mathrm{Ca}}^{2+}+2{\mathrm{HCO}}_{3}^{-}⇋{\mathrm{CaCO}}_{3}+{\mathrm{CO}}_{2}+H_{2}O.$$

Input of alkalinity from the modern mid-ocean ridge is negligible (47). Off-axis carbonation of the oceanic crust has been proposed as a potential sink for ocean alkalinity (48). However, comparisons among drill sites reveal that this carbonation flux is only a small fraction of the river flux and its magnitude still highly uncertain (49). Moreover, the δ^13^C values of carbonate veins in altered oceanic crust are indistinguishable from those of marine carbonate sediments (50). It is therefore reasonable to treat the carbonation flux as part of the broader sedimentary flux, allowing it to be excluded from further consideration in subsequent analyses.

The superplume scenario relies on two main conditions affecting alkalinity: 1) the early release of ~10^6^ PgC to the atmosphere during the orogenic event, and 2) the smaller subaerial land area at 2.2 Ga. Phosphorus levels in ancient sediments suggest continents covered only ~25% of their present extent (51), implying a runoff alkalinity flux of ~0.25 × 3 × 10^12^ eq/y (43).

Because burial of one mole of carbonate consumes two equivalents of alkalinity, sequestering the volcanic carbon would take ~220 Myr; if 30% were buried as organic carbon, this drops to ~155 Myr. These timescales align with the known duration of the LJE (249 ± 9 to 128 ± 9.4 Ma) (8). For comparison, the paleocene-eocene thermal maximum released 2,000 to 7,000 PgC—less than 1% of the CO_2_ attributed to the Birimian superplume. The critical factor is that mantle CO_2_ emissions outpaced the alkalinity supply from weathering over 100 to 200 Myr.

### Carbon Isotope Effects.

Organic carbon burial is often considered the primary driver of long-term δ^13^C variability, but reanalyses show that large burial shifts are not required. Changes in ocean circulation, biological pump efficiency, and air–sea CO_2_ exchange can account for much of the variability.

The carbon isotope mass balance is partitioned among carbonate species:

[2] $$\begin{array}{c} \delta^{13}C_{\mathrm{sw}}=\frac{\left[H_{2}{\mathrm{CO}}_{3}\right]}{\mathrm{DIC}}\delta^{13}C_{H_{2}{\mathrm{CO}}_{3}}+\frac{\left[{\mathrm{HCO}}_{3}^{-}\right]}{\mathrm{DIC}}\delta^{13}C_{{\mathrm{HCO}}_{3}^{-}}\\ +\frac{\left[{\mathrm{CO}}_{3}^{2-}\right]}{\mathrm{DIC}}\delta^{13}C_{{\mathrm{CO}}_{3}^{2-}}, \end{array}$$

Using the fractionation factors of Zeebe & Wolf-Gladrow (46) at 20 °C, which we assume are a reasonable assumption for the early Paleoproterozoic ancient ocean:

[3] $$\delta^{13}C_{\mathrm{dol}}=\delta^{13}C_{\mathrm{sw}}+2+10.7\frac{\left[H_{2}{\mathrm{CO}}_{3}\right]}{\mathrm{DIC}}+\frac{\left[{\mathrm{HCO}}_{3}^{-}\right]}{\mathrm{DIC}}.$$

This emphasizes that isotopic fractionation depends on carbonate speciation in surface waters, controlled by alkalinity and pCO_2_. Since [H_2_CO_3_] ≈ 0.034 × *p*CO_2_ (in atm), the modern ocean—with low pCO_2_ and high alkalinity—keeps [H_2_CO_3_] minimal.

Under greenhouse conditions, higher pCO_2_ and weathering increase erosion but also lower alkalinity flux by enhancing CaCO_3_ precipitation and CO_2_ loss in rivers. As alkalinity and [HCO_3_^−^] fall, more DIC shifts to H_2_CO_3_, and total DIC becomes limited by CO_2_ solubility. Thus, elevated δ^13^C in carbonates (dolomite, calcite) is a diagnostic signal of high-pCO_2_, low-alkalinity states.

### Oxygen Isotope Effects.

Isotopic data (S, Fe, Mo) from the Francevillian Basin have been interpreted as evidence for widespread oxygenation during the LJE (11). Our observation of coupled δ^13^C–δ^18^O bimodality reinforces the view that Earth’s oxygen cycle was profoundly disrupted at this time, possibly linked to a shift in the Dole Effect.

The Dole Effect—the enrichment of atmospheric O_2_ in ^18^O relative to seawater—arises because photosynthesis produces ^18^O-depleted oxygen, while respiration preferentially consumes ^16^O. Their imbalance, especially in a biosphere with substantial terrestrial productivity, drives atmospheric O_2_ toward heavier isotopic values (52, 53). Retallack and Bindeman (54) show that the isotopic offset between marine and continental carbonates (δ^13^C, δ^18^O, Δ′^17^O) increases with the expansion of land-based life, supporting a link to rising continental productivity. An unresolved issue with the preferential expansion of land-based life is that the terrestrial biomass represents a major phosphorus reservoir, thereby reducing phosphorus fluxes to the oceans and ultimately lowering marine productivity.

On shorter timescales, ice-core and speleothem records demonstrate that atmospheric δ^18^O covaries with pCO_2_ and climate at orbital (Milankovitch) frequencies (55, 56). Large volcanic CO_2_ emissions may have further perturbed this balance by modifying oxygen production, consumption, or atmospheric mixing.

Finally, evidence indicates that oxygenic photosynthesis on land predated the permanent rise of atmospheric oxygen (57). Microbial mats on soils and surfaces likely generated localized “whiffs” of oxygen, rapidly consumed but still leaving oxidation signatures in the rock record. This helps explain oxygen-dependent processes in a largely anoxic atmosphere.

### Importance for Early Life.

The early Birimian basaltic eruptions injected large volumes of volcanic CO_2_ into the atmosphere, initiating a greenhouse pulse. This was followed by arc-related magmatism, which oxidized most residual reduced mantle carbon, further intensifying greenhouse conditions When combined with rising atmospheric oxygen and the emergence of substantial land areas above sea level, this created conditions that reshaped Earth’s surface environments in four key ways:

1. Enhanced weathering from pyrite oxidation

The GOE intensified chemical weathering of continental crust through pyrite oxidation (58). Although atmospheric oxygen began increasing around 2.425 Ga (6), sustaining a steady nutrient supply until the end of the Huronian period [ca. 2240 Ma, (6)] likely required additional mechanisms—such as oscillations in atmospheric oxygen and repeated glacial cycles that refreshed pyrite surfaces or the substantial SO_2_ emissions from Birimian volcanism that accelerated weathering.

2. Increased alkalinity and nutrient delivery

Silicate weathering boosted both ocean alkalinity and the flux of phosphorus and trace metals. This is reflected in δ^13^C-enriched stromatolitic carbonates from the Tulomozero Formation (Russia) (59) and the FC Formation (Gabon) (60), both deposited in shallow-marine environments during or just after the LJE.

3. Shift in ocean chemistry from runoff

Erosion of uplifted terrains increased runoff of freshwater rich in major cations but low in Fe^2+^. Mixing with hydrothermal fluids shifted the dominant marine sink from iron formations to carbonate precipitation. In Gabon, the FA Formation—just below the black shales of the FB Formation—hosts the Oklo natural reactors. There, uranyl carbonates transported by O_2_- and CO_2_‚-rich runoff precipitated uranium oxides in the presence of organic matter, indicating vigorous chemical weathering and nutrient transport.

4. Localized nutrient enrichment and eukaryotic potential

Increasing fluxes of nutrients, and notably phosphate—eroded from the reliefs of the nearby Birimian protocontinent—and desorbed from kaolinite and smectite during flocculation of suspended sediments (61) may have created nutrient-rich microenvironments favorable for early eukaryotic life. Centimeter-sized lenticular forms found in the black shales of the FB Formation (Fig. 1), previously interpreted as biological based on their morphology and Zn concentration and isotope data (14), remain under debate as to their potential biogenic identity. While this study does not confirm their biological origin, it provides a compelling environmental framework in which early eukaryotic innovation could plausibly occur, especially in the vicinity of the unique neighboring Oklo natural reactors, which would have produced heat to further intensify nutrient leaching from sediments to feed early eukaryotes.

## Materials and Methods

After leaching the whole-rock sample powders in hot 6 M distilled HCl prior to digestion in a 3:1:0.5 mixture of concentrated distilled HF:HNO_3_:HClO_4_, lead was separated and purified by anion-exchange (BioRad AG1-X8, 100-200 mesh; 0.5 mL resin bed volume) column chromatography using 1 M distilled HBr to elute the sample matrix and 6 M distilled HCl to collect the Pb. Lead was subsequently analyzed for its isotopic composition on a Nu Plasma 500 HR multiple-collector inductively coupled plasma mass spectrometry (MC-ICP-MS) at the Ecole Normale Supérieure de Lyon.

In this work, the thallium technique commonly used for Pb isotope analysis by MC-ICP-MS (62) was replaced by sample-standard bracketing. Faraday-cup efficiencies were computed exactly with as little deviation from unity as possible by repeatedly running the Pb standard solution NIST 981 in alternation with samples. The 2-sigma uncertainties evaluated by an autoregressive filter on the NIST 981 Pb standard as measured in Lyon by MC-ICP-MS are 250 ppm for ^204^Pb/^206^Pb and 75 ppm for ^207^Pb/^206^Pb.

## Supplementary Material

Appendix 01 (PDF)

Dataset S01 (XLSX)

## Data Availability

All data are included in the manuscript and/or supporting information.
