# Supplementary material for: Volcanic forcing of the Lomagundi–Jatuli carbon isotope excursion
Source: Proc Natl Acad Sci U S A. 2025 Dec 1;122(49):e2519431122. doi: 10.1073/pnas.2519431122 (PMC12704732; doi:10.1073/pnas.2519431122)
Supplement: Supplementary file 1 — Appendix 01 (PDF) [file pnas.2519431122.sapp.pdf]

PNAS

Supporting Information for
Volcanic Forcing of the Lomagundi-Jatuli Carbon Isotope Excursion

Janne Blichert-Toft[1], Kurt Konhauser, Baptiste Coutret, Marine Pinto, Arnaud Agranier, Abderrazzak El Albani, and Francis Albarède[2]

[1]Co-Corresponding author: Francis Albarède
Email: albarede@ens-lyon.fr
[2]Co-Corresponding author: Janne Blichert-Toft
Email: jblicher@ens-lyon.fr

This PDF file includes:

Supporting text
Figure S1
Table S1
Dataset S1 (provided as a separate file)
SI References

[1] Co-Corresponding author : Janne Blichert-Toft, ENS de Lyon (LGL-TPE), 9 rue du Vercors, 69007 Lyon, France jblicher@ens-lyon.fr
[2] Co-Corresponding author : Francis Albarede, ENS de Lyon (LGL-TPE), 9 rue du Vercors, 69007 Lyon, France albarede@ens-lyon.fr

Supporting Information Text

Previous geochronology work on the Francevillian of Gabon. The age of 2083 Ma suggested by Horie et al. (1) as the minimum age of the Francevillian series rests on a single zircon from a zircon population extracted from a distant cinerite and ignimbrite with unmistakable detrital contributions.

Leachate-residue mineral and whole-rock Pb-Pb data on black shales from the Francevillian FB series give old ages of 2235 and 2328 Ma (2), but, because of the narrow spread in the Pb isotope compositions of this sample suite, the ages remain too imprecise for a useful assessment of the age of the FB series and, hence, the local LJE.

As suggested by the scatter of Gauthier-Lafaye et al.'s (2) analyses, the significance of the 1.89 Ga Pb-Pb age obtained from a whole-rock isochron by El Albani et al. (3) on the Francevillian FB lenticular forms and their black shale host rocks is impaired by the lack of sample leaching. This work was focused primarily on Zn isotope analysis, which may be affected to an unknown extent by preferential removal of acid-soluble mineral phases, therefore requiring that acid leaching of samples analyzed for Zn isotope compositions be avoided. While Zn is firmly held by mineral and organic phases, the young Pb-Pb age of this study may reflect subtle Pb isotope perturbation by late diagenesis.

Bros et al. (4) determined two internal 147Sm-143Nd isochrons on black shales with ages of 2099 ± 115 Ma and 2036 ± 79 Ma. As attested to by rare-earth element isotopic anomalies in apatite from these samples (5 , 6), the accuracy of the Sm-Nd ages is limited by the inevitable effect of fluid circulation around the Oklo natural reactor, which was alive two billion years ago, due to the strong temperature gradients brought about by radiation. Unknown amounts of Sm with isotope compositions altered by ^{235}U fission in the Oklo natural reactor was undoubtedly present within and in the vicinity of the reactor (7-9). The samples analyzed for Sm-Nd chronology by Bros et al. (4) were spiked, but their $^{147}Sm/^{144}Nd$ ratios before spike addition were not measured. The ^{147}Sm-^{143}Nd ages obtained from the internal isochrons are therefore uncertain and potentially inaccurate.

The Monte Carlo method. The Monte Carlo (MC) method is a well-established approach first introduced by Metropolis and Ulam (10). Its applications in simulation and modeling are extensively covered in more recent literature (11, 12). Multiple algorithms exist for calculating least-squares straight-line fits. In the early days of least-squares fitting of straight lines, the computational cost of MC-based error propagation was prohibitive, leading to a preference for iterative approximations based on first-order expansions (13-15).

One issue is finding the 'best' age and the consensus on the result is good. Several algorithms exist for calculating such least-squares straight-line fits. One approach favors York's cubic method, while other groups may prefer the

Williamson linear algorithm. Our analysis employs software based on the principles introduced by Williamson and implemented by Minster et al.(16). This approach is essentially equivalent to the Newton-Raphson technique developed by Kent et al. (17).

For the second issue, error propagation and statistical test, our software implements an MC-based approach originally introduced by Minster et al. (16) and later integrated by Ludwig as an option in Isoplot (18). The advent of modern desktop computing has now made MC simulations the preferred method, effectively rendering standard first-order expansion techniques obsolete. The MC method offers a more comprehensive and flexible representation of uncertainties by accommodating a wide range of error distributions, an advantage not provided by conventional algorithms. In the 2008 Isoplot 3.7 manual, Ludwig states: "The most reliable errors are obtained with a Monte Carlo simulation that involves the regression procedure itself, and so does not rely on the usual first-derivative expansion approximations at any stage." This methodological choice is particularly well-suited to Pb isotope geochemistry, where errors are small and exhibit strong correlations. It must be emphasized that an independent source of uncertainties, mass-dependent fractionation, whether analytical or natural, is not dealt with by any of these algorithms. As a consequence, the use of the MSWD should be, at least for testing the statistical significance of Pb-Pb alignments, be considered with caution.

Heading

Ocean behavior when pCO_2 increases and Alkalinity → DIC. When dissolved inorganic carbon (DIC) reaches its maximum value, equal to alkalinity, the ocean's buffering system becomes ineffective. If pCO_2 continues to increase – for example, due to a volcanic eruption – the ocean's ability to absorb more CO_2 is significantly diminished. In such conditions, the ocean may start releasing CO_2 instead of taking it up.

Carbonic acid (H_2CO_3) is directly related to pCO_2, as described by Henry's Law:
$[CO_2(aq)] = K_0 \times pCO_2$
where:
- K_0 is the solubility constant (~0.034 mol/(L·atm) at 25°C).
- pCO_2 is the partial pressure of carbon dioxide.

When DIC equals alkalinity, ions CO_3^{2-} are entirely depleted. As a result, additional CO_2 dissolves primarily as carbonic acid:
$CO_2(aq) + H_2O \rightleftharpoons H_2CO_3$
Without carbonate ions to neutralize the hydrogen ions produced, *p*H decreases and surface pCO_2 increases in step with atmospheric pCO_2.

Ocean buffer saturation: why CO_2 uptake stops

When carbonate ions are depleted, the buffering reaction that helps regulate ocean pH is inhibited:
$CO_3^{2-} + H^+ \rightarrow HCO_3^-$
As this occurs:
- Carbonate ions disappear, reducing the ocean's ability to absorb CO_2.
- Additional CO_2 dissolves, raising surface pCO_2 without providing meaningful pH stability.

The system moves from acting as a CO_2 sink to a CO_2-neutral state and eventually becomes a CO_2 source.

The critical turning point: outgassing begins
If atmospheric pCO_2 continues to rise—such as during a volcanic eruption—surface waters can become supersaturated with CO_2. When:
pCO_2(ocean surface) > pCO_2(atmosphere),
the ocean starts to release CO_2:
$CO_2(aq) \rightarrow CO_2(g)$
At this point, the ocean stops absorbing CO_2 and instead begins emitting it into the atmosphere, reversing its usual role as a carbon sink.

Effect of large pulses of volcanic CO2 on the pH
Once pCO_2 exceeds ~0.1 atm, i.e., the conditions for which DIC = Alk (e.g., (19)).

$$\mathrm{pH} = \mathrm{pK_1} + \log_{10}\frac{[HCO_3^-]}{[H_2CO_3]} = 7.57 + \log_{10}(\mathrm{Alk}) - \log_{10}(\mathrm{pCO_2})$$

(Henderson-Hasselbalch equation), where K_1 is the acid dissociation constant for carbonic acid (pK1~6.1 in seawater). For high pCO_2 values (≥1 atm), the carbonate system becomes chemically unsustainable. The ocean would either:

1. Outgas CO_2 to restore equilibrium.
2. Shift carbonate equilibria via dissolution of carbonate sediments.
3. Experience severe acidification beyond the buffering range.

The ocean's carbonate buffering system is completely overwhelmed and the pH cannot remain stable (Fig. S1).

The role of temperature: amplifying the effect
Warming surface waters can accelerate this transition. Since CO_2 solubility decreases as temperature increases since CO_2 solubility ∝ 1/T. Rising ocean temperatures cause more CO_2 to escape, amplifying the greenhouse effect.

Real-world scenario: volcanic CO_2 injection
A volcanic eruption can inject large quantities of CO_2 into the atmosphere. If DIC has already reached the alkalinity limit, the ocean's capacity to absorb additional CO_2 is overwhelmed, resulting in:
- CO_2 supersaturation near the surface.
- Elevated surface pCO_2 and increased acidification.

- Outgassing of CO_2 back into the atmosphere.

The carbon isotope effect

Let us write the isotope mass balance as:

$$\delta^{13}C_{sw} = \frac{[H_2CO_3]}{DIC}\delta^{13}C_{H_2CO_3} + \frac{[HCO_3^-]}{DIC}\delta^{13}C_{HCO_3^-} + \frac{[CO_3^{2-}]}{DIC}\delta^{13}C_{CO_3^{2-}}$$

Subtracting $\delta^{13}C_{CO_2(g)}$

$$\delta^{13}C_{sw} - \delta^{13}C_{CO_2(g)} = \frac{[H_2CO_3]}{DIC}\Delta\delta^{13}C_{H_2CO_3} + \frac{[HCO_3^-]}{DIC}\Delta\delta^{13}C_{HCO_3^-} + \frac{[CO_3^{2-}]}{DIC}\Delta\delta^{13}C_{CO_3^{2-}}$$

in which Δ's refer to the difference in $\delta^{13}C$ with respect to gaseous CO_2. Using Zeebe and Wolf-Gladrow's equations (Table S1) to get the Δ values at 20°C, and the definition

$$\delta^{13}C_{CO_2(g)} = \delta^{13}C_{dol} - 11.6$$

(dol=dolomite) we get:

$$\delta^{13}C_{sw} = \delta^{13}C_{dol} - 11.6 + \frac{[H_2CO_3]}{DIC}(-1.1) + \frac{[HCO_3^-]}{DIC}(+8.6) + \frac{[CO_3^{2-}]}{DIC}(+9.6)$$

which can be rearranged as:

$$\delta^{13}C_{sw} = \delta^{13}C_{dol} - 11.6 + \frac{[H_2CO_3]}{DIC}(-1.1) + \frac{[HCO_3^-]}{DIC}(+8.6) + \left(1 - \frac{[H_2CO_3]}{DIC} - \frac{[HCO_3^-]}{DIC}\right)(+9.6)$$

or

$$\delta^{13}C_{dol} = \delta^{13}C_{sw} + 2 + 10.7\frac{[H_2CO_3]}{DIC} + \frac{[HCO_3^-]}{DIC}$$

which shows that lower alkalinity and higher pCO_2 increase dolomite-seawater $^{13}C/^{12}C$ fractionation.

An important question is: can f_{org} change if the $\delta^{13}C$(input) remain constant? Let us breakdown the mass balance at steady-state:

$$\delta^{13}C(\text{input}) = f_{org}\delta^{13}C(\text{org}) + f_{carb}\delta^{13}C(\text{carb}) + rf_{carb}\delta^{13}C(CO_2)$$

and eliminate fcarb:

$$\delta^{13}C(\text{input}) - \frac{r}{1+r}\Delta\delta^{13}C(CO_2 - \text{carb}) = ff_{org}\,\delta^{13}C(\text{org}) + (1 - f_{org})\,\delta^{13}C(\text{carb})$$

Using Table S1, this can be finally simplified at 20°C as:

$$\delta^{13}C(\text{input}) - 4.35 = \delta^{13}C(\text{carb}) + f_{org}\left[\delta^{13}C(\text{org}) - \delta^{13}C(\text{carb})\right]$$

in which we recognize the standard mass-balance equation shifted by 4.35‰ to account for CO_2 outgassing. For the $\delta^{13}C$(input) value to remain constant, the difference between $\delta^{13}C$(org) and $\delta^{13}C$(carb) must remain constant.

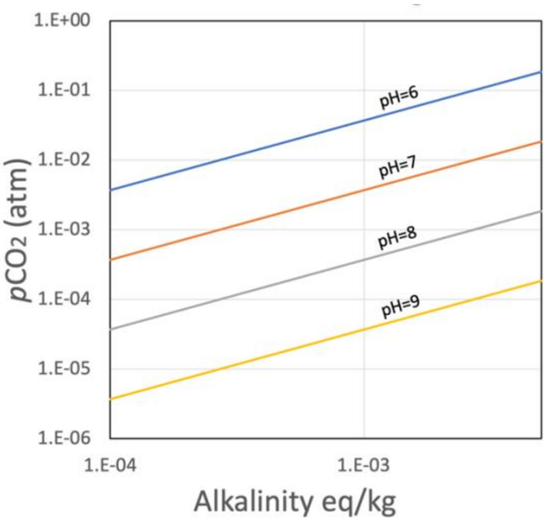

Fig. S1. Relationship between pCO_2 and Alk for different pH values. The modern value of seawater alkalinity is ca. 2.4 mmol/kg (2.4 meq/L).

Table S1. Isotopic fractionation of carbon ($\Delta\delta$13C, in ‰) relative to CO_2 (gas) calculated from the equations in ref (20).

Temp. (°C)	H_2CO3	HCO_3^-	CO_3^{2-}	Calcite	Dolomite
0	-1.1	10.5	11.5	13.2	13.5
5	-1.1	10.0	11.0	12.7	13.0
10	-1.1	9.5	10.5	12.2	12.5
15	-1.1	9.0	10.0	11.7	12.0
20	-1.1	8.6	9.6	11.3	11.6
25	-1.1	8.2	9.2	10.9	11.2

Dataset S1. Pb isotope data for carbonate samples from the study: "*Volcanic Forcing of the Lomagundi–Jatuli Carbon Isotope Excursion*"

(Provided as a separate Excel file: Dataset S1.xlsx)

SI References

1. K. Horie, H. Hidaka, F. Gauthier-Lafaye, Elemental distribution in zircon: alteration and radiation-damage effects. Physics and Chemistry of the Earth, Parts A/B/C **31**, 587-592 (2006).
2. F. Gauthier-Lafaye, R. Bros, P. Stille, Pb isotope systematics on diagenetic clays: an example from proterozoic black shales of the Franceville basin (Gabon). Chemical Geology **133**, 243-250 (1996).

3. A. El Albani et al., A search for life in Palaeoproterozoic marine sediments using Zn isotopes and geochemistry. Earth and Planetary Science Letters **612**, 118169 (2023).
4. R. Bros, P. Stille, F. Gauthier-Lafaye, F. Weber, N. Clauer, Sm-Nd isotopic dating of Proterozoic clay material: An example from the Francevillian sedimentary series, Gabon. Earth and Planetary Sctence Letters **113**, 207-218 (1992).
5. R. Bros, J. Carpena, V. Sere, A. Beltritti, Occurrence of Pu and fissiogenic REE in hydrothermal apatites from the fossil nuclear reactor 16 at Oklo (Gabon). Radiochimica Acta **74**, 277-282 (1996).
6. K. Horie, H. Hidaka, F. Gauthier-Lafaye, Isotopic evidence for trapped fissiogenic REE and nucleogenic Pu in apatite and Pb evolution at the Oklo natural reactor. Geochimica et Cosmochimica Acta **68**, 115-125 (2004).
7. M. Loubet, C. Allegre, Behavior of the rare earth elements in the Oklo natural reactor. Geochimica et Cosmochimica Acta **41**, 1539-1548 (1977).
8. P. Holliger, C. Devillers, Contribution of the measurement of lutetium isotope ratio to the temperature study of the Oklo reactor. Earth and Planetary Science Letters **52**, 76-84 (1981).
9. H. Hidaka, F. Gauthier-Lafaye, Redistribution of fissiogenic and non-fissiogenic REE, Th and U in and around natural fission reactors at Oklo and Bangombé, Gabon. Geochimica et Cosmochimica Acta **64**, 2093-2108 (2000).
10. N. Metropolis, S. Ulam, The monte carlo method. Journal of the American statistical association **44**, 335-341 (1949).
11. R. Y. Rubinstein, D. P. Kroese, Simulation and the Monte Carlo method (John Wiley & Sons, 2016).
12. J. Hammersley, Monte carlo methods (Springer Science & Business Media, 2013).
13. D. York, Least-squares fitting of a straight line. Canad. J. Phys. **44**, 1079-1089 (1966).
14. D. York, Least squares fitting of a straight line with correlated errors. Earth Planet. Sci. Letters **5**, 320-324 (1969).
15. J. H. Williamson, Least-squares fitting of a straight line. Canad. J. Phys. **46**, 1845-1847 (1968).
16. J.-F. Minster, P. Ricart, C. J. Allègre, ^{87}Rb-^{87}Sr geochronology of enstatite meteorites. Earth Planet. Sci. Letters **42**, 333-347 (1979).
17. J. T. Kent, G. S. Watson, T. C. Onstott, Fitting straight lines and planes with an application to radiometric dating. Earth Planet. Sci. Letters **97**, 1-17 (1990).
18. K. Ludwig, Isoplot/Ex, v. 3.75, Berkeley Geochronol. Center, Spec. Publ **5** (2012).
19. M. Frankignoulle, A complete set of buffer factors for acid/base CO2 system in seawater. Journal of Marine Systems **5**, 111-118 (1994).
20. R. E. Zeebe, D. Wolf-Gladrow, CO_2 in seawater: equilibrium, kinetics, isotopes (Gulf Professional Publishing, 2001).
